# Supplementary material for: A comparison of prospective space-time scan statistics and spatiotemporal event sequence based clustering for COVID-19 surveillance
Source: PLoS One. 2021 Jun 10;16(6):e0252990. doi: 10.1371/journal.pone.0252990 (PMC8191960; doi:10.1371/journal.pone.0252990)
Supplement: Attachment — Submitted filename: Response to reviewers.docx [file pone.0252990.s006.docx]

**Response to reviewers**

Reviewer #1: The authors of the study used prospective space-time scan statistic on number of positive COVID-19 cases in counties to determine location and period of most likely space-time clusters in the continental US. Case counts from counties in most likely clusters were then analyzed through hierarchical agglomerative clustering method to further characterize groups of counties with similar patterns in how frequency of cases (standardized by population size) develop over time. Demographic factors were then used in order to evaluate any differences between clusters. Researches reported that counties detected in the same space-time clusters were classified into different hierarchical clusters. This is interesting finding, and a topic worthy of investigation; although not completely surprising. When methods are combined, this could be done in many different ways and authors used one possible approach - at the very end of the manuscript, they offered some alternatives. This alternative approach was something that I was wondering from the very beginning (no actionable comment here).


In my opinion, by far the largest limitation of the manuscript is its length. Manuscript with 38 pages of text (including tables) is more a technical report than a classical journal article. This is clearly a consequence of thorough analysis, but reading of such contribution is very demanding on the reader, particularly when a lot of technical results consists of reporting counties in most likely clusters. This was done for different time periods, followed by hierarchical clustering. Of course, number of clusters in spatial analysis and in multivariate analysis will be different and this further complicates reading. Is it possible to present an analysis for only one time period (the one authors feel is the most informative), and for the rest of results to be offered in the supplementary material? With a short commentary point on how are other results similar or dissimilar? Also, in multivariate cluster analysis, it is often attempted to explain nature of clusters identified. Although this was attempted to some degree here, this information was diluted among many other details. We recognize that there was too much redundancy and detailing of clusters in the initial manuscript. We did consider a focus on a single study period, but we feel there are some interesting comparisons to convey over the four periods. To be responsive to the reviewer’s concern of length and redundancy we have substantially simplified the results section to focus on a more directed comparison of the two approaches.

Because of manuscript length, it is easy to miss some technical details.
For example.
L104 - are the authors suggesting that RR is available for each location in the cluster? I am not sure that this is in line with how scan statistics work. The RR risk is estimated for the population in the scanning window, or maybe I missed the point here.

SatScan does compute the RR for individual locations within a cluster as well as the cluster RR. The RR for a county within a cluster is calculated using the following equation as used in Hohl et al 2020

${RR}_{cty}=\frac{c/e}{(C-c)(C-e)}$where c= total number of cases in a county, C is the total number of observed cases in the conterminous US, *e* is the expected number of cases in a county calculated as $e=p_{cty}*\frac{C}{P}$

L116. Is it possible to determine false positives based on results from scan statistics? Could RR<1 be indication of cluster of cases with lower than expected risk, rather than false positive?

A RR <1 would not be considered a false positive in the sense of the RR for the cluster. Our thinking here is that in the context of a detected cluster, a specific county within the cluster with a county RR <1 might be construed as a false positive. We have however changed the text to avoid any misrepresentation in this regard.

L121. Perhaps check wording. Is it all counties with high RR or all counties from significant clusters with high RR? Individual counties with RR>1 computed as indicated above were used in the STES analysis.

L159. Is "severe" the best adjective to use. severe typically refers to clinical expression, whereas cases are just reflection of incidence, some are likely asymptomatic. We agree that this term is not appropriate here and have removed this text.

L163. Authors should perhaps explain whether they used Poisson model or space-time permutation model. They are different methods. The prospective Poisson space-time models was used. We apologize for the oversight and ambiguity.

L163. In this section, it would also be useful to explain how is prospective statistic different from retrospective which is commonly applied in retrospective research studies. A more specific distinction between them has been added to the text.

In the methods section - authors did not mention division into different time periods, and what was the rationale for that. reader learns about that in the results section. In the revised manuscript we have explicitly noted the study time period in the Methods section with some justification for these period intervals.

L239. "spread" may not be the best word to use here. Clusters do not spread. We agree and have changed the text to - as the drivers for these identified clusters.

Throughout the manuscript, some wording should be improved. e.g. L384 writes about 4 statistically significant counties. is it counties or clusters that are significant? We agree and have removed the incorrect reference to statistically significant counties.

In addition, the authors try to compare demographics across different clusters, but are not trying to do any statistical testing which could be helpful as a decision point. Is there a reason for that?

Are the authors concerned that many comparisons could lead to some false findings? In the revisions to the manuscript, we have removed the demographic analysis section as suggested by reviewer 2.

Reviewer #2: Manuscript #: PONE-D-20-26698

Title: Space-time surveillance of COVID-19 emerging hotspots using prospective scan statistics enhanced by spatiotemporal event sequence based clustering.

Authors: Fuyu Xu & Kate Beard

General comments:

Xu and Beard have used an innovative approach to classifying the temporal patterns of “epidemic curves” at the county level that could make a very important contribution to the epidemiological and disease surveillance literature. Unfortunately, the current manuscript suffers from too much repetition, taking on too many objectives, and perhaps taking a less than ideal approach for integrating their event sequence based clustering with scan statistics. Specifically, the following should be addressed:

We recognize that there were too many objectives, too much redundancy and detailing of clusters in the initial manuscript. We did consider a focus on a single study period, but we feel there are some interesting comparisons to convey over the four periods, so we have retained the four study period comparison. To be responsive to the reviewer’s concern of length and redundancy we have substantially simplified the results section to focus on a more directed comparison of the two approaches.

1. While there are a number of good reasons to conduct prospective scans, in the context of this manuscript repeating the analysis several times for different periods during the pandemic in the contiguous US states makes the manuscript exceedingly repetitive. I would recommend the authors pick a single period for the analysis and use it consistently throughout the manuscript to exemplify their approach. We have revised the manuscript to remove the redundancies of describing each period in detail. We feel that the comparison between the space-time scan and the sequence similarity clusters benefits from a comparison over a sequence of time periods. Thus, we have retained the 4 study periods but limit the results and discuss to key comparisons of what is conveyed in the space-time scan view versus the temporal view conveyed by the sequence similarity.

2. In terms of the use of the scan statistic, there are a number of major issues that should be addressed by the authors:

i. The decision to limit the maximum size of a cluster to 10% of the population appears to be arbitrary. I would recommend using the default of 50% or less (also the maximum that can be used) of the population. This value does not prevent smaller clusters from being detected, but prevents the need for arbitrary values and is the whole point of the flexible scanning window. In reviewing the figures, it is clear that during certain periods a large number of small clusters are likely part of a larger cluster. Determining a specific upper bound of scanning window size has been explained in papers by Kulldorf et al. and SaTScan User Guide 9.6. The optimum maximum size of the scanning window should be determined on case by case. 10% of the population was used in similar research (Holz et al 2020) and it seemed is reasonable to replicate that here. We did experiment with using 50% of population for an upper limit which resulted in some extremely large clusters especially at the early stage of pandemic.

ii. The authors need to clearly state what rule was used for reporting space-time clusters in terms of spatial overlap and preferably spatio-temporal overlap. There is no spatial overlap in clusters in the output results, but temporal overlaps do occur.

iii. The authors are unclear whether they are using a Poisson model or the space-time permutation model for their space-time scans. On lines 166-167, they refer to the permutation model and later to the Poisson distribution on line 185. The space-time permutation model is not based on a Poisson distribution and only requires case data. The prospective Poisson space-time model was used. We apologize for the oversight and ambiguity and have made clarifications in the text.

iv. If the authors used a Poisson model in SaTScan, it is unclear why they did not adjust for age and sex; this type of standardization is common in most epidemiological analyses. If they used a space-time permutation model, they should recognize that these models identify space-time clusters while inherently adjusting for purely spatial and temporal clusters; in other words this model would adjust for demographic and socio-economic factors if they were related to spatial location. We did not have information on age and sex for confirmed cased so were not able to make these adjustments. We also note in the text that while deaths from COVID 19 are several times higher in older age groups, infections can affect all segments of the population.

3. It is unclear why the authors did not apply their event sequence based clustering to all counties. The value seems to be diminished by only applying the technique to locations within active space-time clusters. In fact, investigating if there were spatial clusters of these event sequence clusters, using a multinomial model, would have been a very interesting and perhaps a more appropriate way to combine the two approaches and comment on whether or not these event sequence clusters were randomly distributed or had particular “hotspots”. It would certainly be interesting to compare space-time “hotspots” for rates of disease with spatial clusters of these event sequence clusters. We did apply the sequence similarity-based clustering to all counties, but in this early period of the pandemic many counties had no cases. We have now included these in one “Outsiders” category in the temporal profiles that have been added to sequence similarity cluster maps. As an objective was to compare differences between space-time scan and sequence similarity clusters, we also felt it was useful to focus on the most active case locations. While some of the sequence similarity clusters exhibit some spatial clustering, an aim of the sequence similarity approach was to offer a temporal view on the locations identified by the space-time scan.

4. The event sequence clusters shared in geographically distant regions (e.g., Pacific Northwest and New York) likely reflect when COVID-19 was introduced into the US even though molecular sequencing suggests the introduction in these regions was likely from different continents of origin (i.e., Europe vs. Asia). Some discussion concerning the epidemiological interpretation of clusters based on the greater literature is warranted. We have added references to research on the different continents of origin noting that isolates from China primarily seeded the original COVID-19 outbreak on the West Coast and that European isolates seeded the pandemic in New York (and the US East Coast).

5. The socio-demographic analyses should probably be removed from this manuscript. I would recommend performing these analyses using multivariable multinomial regression models based on the event sequence cluster classification for each county in a separate manuscript. Currently, the descriptive comments concerning socio-demographic factors within clusters identified using different methods are not particularly insightful in the current draft of the manuscript. I suspect the authors have put too much in one manuscript and this section deserves much more detail and a stronger analysis. We agree that this analysis would be better served in another manuscript and have removed it.

Specific comments:

Title:
i. The event sequence based clustering was applied to counties during a specific time period, but it did not account for spatial location. It might be better for the authors to state that they are examining the distribution of counties classified based on event sequence based clustering with respect to space-time clusters of COVID-19. If they agree with comment 3 in my general comments, it might be better to focus on the spatial clustering of event sequence based clusters of COVID-19. We have revised the title to reflect changes to: “A comparison of prospective space-time scan statistics and event sequence similarity based clustering for COVID 19 surveillance.”

Additionally, we have made revisions in the manuscript to clarify that the sequence similarity clustering was applied in each study period, revised the maps of these clusters and added timelines to show the temporal profiles of these clusters. Some of the sequence similarity clusters do exhibit spatial clustering of some members but the intent was to examine membership with respect to sequence similarity rather than spatial clustering.

Introduction:

i. Lines 60-62: This statement is not really correct. Where and when these measures were implemented, there was success in “flattening the curve” (even in the US), and strict measures did control the disease in parts of the world where the political, economic, and sociological conditions allowed for their strict implementation. I would remove this sentence since it is not relevant to the authors’ work or the need for surveillance tools for the continuing pandemic. This text has been removed in the revised manuscript.

ii. Lines 97-98. This statement is not accurate. The space-time permutation model that is available with SaTScan does not use or require background population data although as a result, it is subject to population shift bias. We did not use the permutation space-time scan statistic. The prospective Poisson space-time models was used. We apologize for the ambiguity in line 168 and have corrected this oversight in the revised manuscript.

iii. Lines 108-109. There’s nothing that prevents an individual from making these comparisons. Please note that the point of the scan statistic is to detect if there are clusters in space, time, and space-time with significantly higher or lower levels of disease without predefining the geographical or temporal size of these clusters. There is no implication that every sub-region within a cluster shares the same rates any more than one could assume that the rate of disease among cities, towns, or villages within a county were homogeneous. The text in this section was removed.

iv. Lines 110-129. This reads more like a summary of methods. I would strongly suggest the authors clearly state their research objectives (i.e., what are they trying to discover or compare rather than a brief description of the methods). We have revised this section to specifically address the paper objectives and have moved the methods related text to the Methods section.

Methods:

i. Please review suggestions in the general comments especially concerning the scan statistic.
We have addressed the suggestions in the general comments on the scan statistic.

ii. Lines 156-161. The authors should use appropriate epidemiological terms. The authors need to clearly state they are calculating the crude incidence rate. However, I would strongly encourage them to consider calculating the age and sex adjusted incidence rates for their subsequent event sequence based clustering. We thank the reviewer for this correction and have revised the manuscript to refer to incidence rate. We did not have information on age and sex for confirmed cased so were not able to make these adjustments. We also note in the text that while deaths from COVID 19 are several times higher in older age groups, infections can affect all segments of the population.

iii. Lines 183-184. The authors state that the duration of a cluster was set at 2 days, but in the results they have space-time clusters of 1 day in length. The authors should revise the methods or results for consistency. The setting with 2 days is correct. A date math error occurred when presenting the cluster duration in the table, which we have corrected in the revised manuscript.
iv. Line 188. Please note the number of Monte Carlo replications performed and whether scans were performed as 1-tailed tests needs to be stated. We chose the Standard Monte Carlo for the p-value (<= 0.05) in the SaTScan setting, and 999 simulations were run.

v. Line 198. Please replace the phrase “cases normalized to the county population” with the term incidence rate throughout the manuscript. This has been corrected through-out the revised manuscript.

Results:

i. Please make certain tables of clusters have consistent titles and select one short form to differentiate event sequence based clusters from space-time clusters and use it consistently throughout the manuscript. Please avoid repeating the methods in the results and use subheadings to differentiate the different statistical approaches being used. Avoid including discussion in the results section. We have revised the test to clearly separate results from discussion.

ii. The tables and figures provide a great deal of summary detail. Please use the text to describe general locations and major characteristics of clusters. The listing of each county within a cluster or each county with a high rate of disease in the text is not necessary. For tables of space-time clusters, please include a column for the radius, the column for the log likelihood in unnecessary. Some authors would include latitude and longitude in these tables, but the figures are sufficient for spatial information. We have included a column for the radius and removed the column of log likelihood (LLR).

iii. The figures concerning the “elbow method” and the dendrograms should be moved from the supplemental material into the main manuscript. If the authors follow the suggestions in the general comments, this figure would only be needed for the one period being examined. In the revised manuscript we have moved the figures of the elbow method graphs and cluster dendrograms to the main body.

iv. Line 339. Please remove the term “emerging”. We have removed this term.

v. Line 450. Please note that it is the cluster that is statistically significant and not the counties. Please remove similar phrases from the text. We have corrected this in the revised manuscript.

vi. Line 473. Figures and tables need to be numbered in the order they appear in the text. Currently, figure 9 is listed before figures 7 and 8. We have revised some figures and taken care that they appear in the correct order and in which they are referenced in the text.

vii. Line 522-523. Please remove results that are not statistically significant from the text and figures. We have removed the non-statistically significant clusters in the figures and the corresponding description in the text.

Discussion:

i. Please revise the discussion after revising the manuscript. The discussion has been revised to reflect revisions to the manuscript.

3)  We note that Figures 1- 8 in your submission contain map images which may be copyrighted. With respect to the maps in the manuscript, these were created directly by us using open source geographic county boundary files from the US Census. The maps do not include proprietary basemap images.
