# Supplementary material for: A comparison of prospective space-time scan statistics and spatiotemporal event sequence based clustering for COVID-19 surveillance
Source: PLoS One. 2021 Jun 10;16(6):e0252990. doi: 10.1371/journal.pone.0252990 (PMC8191960; doi:10.1371/journal.pone.0252990)
Supplement: Attachment — Submitted filename: response_Reviewers_v3.docx [file pone.0252990.s007.docx]

**Response to Reviewers**

Reviewer #1: Thank you to authors for addressing points raised in the previous review. I only have a couple of minor suggestions for authors to consider.

Authors stated that incubation is 14 days, which is approaching upper limit. Ideally, the authors should carefully word whether this was maximum or average and provide reference. Thanks for pointing this out. We cited a reference for this in which the incubation time for COVID-19 is mostly ranging from 1-14 days with the average of 5 days.

Next, on images displaying case sequence clusters – it may be useful to indicate, possibly in the figure legend, what are outsiders. We added text in the methods section on comparison to the group outside the clusters which is the Outsiders or OC group. This is also now noted in the captions of all four related maps.

Similarly, for cluster dendrograms it may be helpful to indicate which group of observations belong to which cluster. It seems there is a place for this, but cluster designation is not visible on figures. We added the cluster numbers to the dendrogram figures to facilitate reference between them and the maps. They are also consistently color coded now between figures.

In addition, the authors used calendar time to look into similarity of case incidence within clusters. For future consideration, it may actually be interesting to consider time in terms of number of days since detection of the first case in a county. In this paper we emphasize the purpose of surveillance, so using calendar time seemed appropriate. We agree that using the number of days since detection of the first case in a county for a time measure is an interesting approach for future research. As a matter of fact, we thought this before and believe that using the time counting from the first incidence detection in a county is better for identifying the similar patterns in terms of the development of covid-19 disease itself.

Reviewer #2: Manuscript ID: PONE-D-20-266698R1
Title: A comparison of prospective space-time scan statistics and event sequence similarity-based clustering of COVID-19 surveillance
Authors: Xu, F. & Beard, K.

General comments: The revised draft of the authors manuscript is greatly improved. Although I am not sure I agree with all their decisions (e.g., maximum scanning window), I believe they have documented/defended their decisions well. My remaining suggestions are mainly cosmetic in nature. Below are some general comments/suggestions:
i. Please put subheadings for the space-time cluster and sequence similarity-based cluster paragraphs in each study period section to avoid confusion over what type of “clusters” are being discussed. We added subheading for space-time scan cluster and sequence similarity-based cluster paragraphs for each of the study periods.

ii. Spell “sequence similarity-based cluster” consistently throughout the manuscript. It is written at least three different ways in the text (e.g., “sequence-similarity based cluster”, “sequence similarity based cluster”). Thank you for catching this. We made the consistent use of “sequence similarity-based cluster” throughout the manuscript.

iii. In paragraphs concerning sequence similarity-based clusters, please do not refer to “spatial clusters” of these clusters. It is very confusing to use the term in a non-statistical sense in a manuscript describing two types of statistical clusters. Just indicate that these sequence similarity-based clusters concentrate around particular cities or regions rather than state they form “spatial clusters”. When some members or counties of sequence similarity-based clusters are spatially clustered we changed the expression to “some members within this group appear spatially concentrated or grouped around metropolitan areas …”.

iv. In the discussion, make certain to state clearly the value of extracting the information concerning the sequence similarity-based clusters from within the space-time clusters.? We have added a paragraph to the methods section on comparison to address this issue.

Manuscript text:
i. Line 45: It should read “share a similar”. “share similar” has been replaced with “share a similar”.
ii. Line 109: It should read “understanding disease dynamics”. “understanding of the disease dynamics” has been replaced with “understanding disease dynamics”.
iii. Lines 113 & 386: It should read “complementary” not “complimentary”. “complimentary” has been corrected with “complementary”.

iv. Line 165: It might be better to write “missing” rather than “avoiding”. In this case we did want to avoid very large clusters such as ones covering over a quarter of the country as these are not particularly meaningful as “clusters”

v. Lines 171-173: This statement is not accurate. The age structure of a population will influence disease reporting and the real incidence of disease. The authors should just state they did not have access to age and sex data for cases and were unable to adjust for these variables as they reported in their response letter. This limitation should be addressed in the discussion. We agree the effect of age structure of a population on the actual incidence of disease. We added “we were unable to access age and sex data at this time for cases in this study, thus we did not adjust for age and sex”.

vi. Line 219: Would it be better to state “decreased” rather than “improved”? We made this change

vii. Line 263: It should read, “statistical”. We made this change

viii. Line 333: Please remove the sentence, “In this period, we note little activity on the west coast.” This statement is not correct. The authors did not identify any active space-time clusters during this period, but there was a lot of disease activity on the West Coast. We agree with this and the sentence has been deleted.
ix. Line 347: Replace “covering through” with “ending in”. We replaced “covering through” with “ending in” since followed by May 20, 2020.
x. Line 348: Replace “statistics” with “statistic”. We replaced “statistics” with “statistic”.
xi. Line 362: Replace “determined” with “selected”. “determined” has been replaced with “selected”. (note: “determined” was in Line 372)
xii. Lines 371-372: Make certain to explain what “OC” means in the text. We added a paragraph in the methods section to explain OC and additional added an explanation of “Outsiders” (OC) in the captions of all four related maps.
xiii. Lines 389-390: It might be better to state, “the expected number of cases in space-time based on …...”. The following sentence from 390-391 should be removed since it is redundant with the addition of the above phrase. We added “in space-time” after “the expected number of cases” and deleted the following sentence.
xiv. Line 392: It should read, “at such a location during a period of time.” We replaced “at such location and times” with “at such a location during a period of time”.
xv. Line 393: Replace “temporal dimension” with “temporal pattern” since one could argue the “temporal dimension” is part of the space-time cluster. Good point, we replaced “temporal dimension” with “temporal pattern”.
xvi. Line 400: Rephrase as “we found that in all study periods, similar sequence patterns of COVID-19...”. We agreed that this is a better expression so we replaced “all study periods showed that similar sequences in COVID-19 cases” with “we found that in all study periods, similar sequence patterns of COVID-19 cases”.

xvii. Line 408: Consider including “similar changes in surveillance programs” to the list of reasons explaining these common temporal patterns. We agreed with the reviewer adding this to the list for one of the reasons.
xviii. Lines 413-414: Do the authors mean “counties in New York State”? Please clarify.There is a New York county so we changed the order of the statement to read Bronx, Kings, Queens, New York, and Wassau counties in New York State, for better clarification.
xix. Line 447: It should read, “pathway studies”. We replaced “pathways studies” with “pathway studies”.

Tables and figures:
i. Tables 1-4. In the titles, please replace “SaTScan space-time clusters” with “prospective space-time clusters”. A footnote can be added to the tables stating, “Space-time clusters were identified using the spatial scan statistic with a Poisson model”. We replaced “SaTScan” with “prospective” and placed the “Note: Space-time clusters were identified using the spatial scan statistic with a Poisson model” below the tables as a footnote.
ii. Fig 1. Remove “covering” from the title. We deleted “covering”.
iii. I believe the journal expects supplementary materials to be labeled as “Table S1” and “Fig. S1” rather than “S1 Table” or “S1 Figure”. Please correct accordingly. We double checked the author guidelines which state the following: “You may use almost any description as the item name of your supporting information as long as it contains an "S" and number. For example, “S1 Appendix” and “S2 Appendix,” “S1 Table” and “S2 Table,” and so forth”. So we followed this guideline. However, the way of labeling that the reviewer suggested may also be accepted.

References:
i. Please properly edit the references. Journal titles and manuscript titles are inconsistently formatted. Additional “PubMed” information is sometimes accidentally included at the end of references. If the authors wish to include “doi” information, please include it consistently or not at all. Manually rechecked and properly edited the Endnote references according to PLoSOne author guidelines. Removed “PubMed” and “doi” information from some references and updated and formatted all references consistently.
ii. Reference 36 should be replaced with a more formal reference (e.g., journal article or government report). The previous Ref 36 was removed and replaced with two recent formal references (Ref 36 and 37).
