# Supplementary material for: A comparison of prospective space-time scan statistics and spatiotemporal event sequence based clustering for COVID-19 surveillance
Source: PLoS One. 2021 Jun 10;16(6):e0252990. doi: 10.1371/journal.pone.0252990 (PMC8191960; doi:10.1371/journal.pone.0252990)
Supplement: Attachment — Submitted filename: response_Reviewers_v4_5-20-2021.docx [file pone.0252990.s008.docx]

**Response to Reviewers**

Reviewer #2: Manuscript ID: PONE-D-20-26698R2

Manuscript title: A comparison of prospective space-time scan-statistics and spatiotemporal event sequence-based clustering for COVID 19 surveillance

Corresponding author: Beard-K

General comments:

The authors have made all the requested revisions. Below are some minor edits/suggestions they should consider. There is no need for me to see the manuscript again.

Specific comments:

Line 172: Should it read, “required”?

We agree that “required” should be used here instead of “require”.

Line 174-175: It might sound better to write, “....for cases in this study, so we could not adjust for age and sex.”

(should be in Line 177 followed by the context of Line 174-175) Thanks for the reviewer’s suggestion here so we have changed the original “…for cases in this study, thus for this study we did not adjust for age.” **TO** “....for cases in this study, so we could not adjust for age and sex.”

References:

Fix the formatting of journal titles for references 17, 21, 34 (also the article title).

We have corrected and updated the cited references for the formatting of article and journal titles. The formatting of the article and journal titles in references 17, 21, 34 is now the same as those of other references.
**Note that** even the tracking version may not be marked because this was done automatically with the EndNote.
